# Effect of perfluorocarbon composition on activation of phase‐changing ultrasound contrast agents

**DOI:** 10.1002/mp.15564

**Published:** 2022-03-07

**Authors:** Trevor M. Mitcham, Dmitry Nevozhay, Yunyun Chen, Linh D. Nguyen, Gianmarco F. Pinton, Stephen Y. Lai, Konstantin V. Sokolov, Richard R. Bouchard

**Affiliations:** ^1^ Department of Imaging Physics University of Texas MD Anderson Cancer Center Houston Texas USA; ^2^ MD Anderson Cancer Center UTHealth Graduate School of Biomedical Sciences Houston Texas USA; ^3^ Department of Head and Neck Surgery University of Texas MD Anderson Cancer Center Houston Texas USA; ^4^ Joint Department of Biomedical Engineering University of North Carolina at Chapel Hill and North Carolina State University Chapel Hill North Carolina USA; ^5^ Department of Bioengineering Rice University Houston Texas USA; ^6^ Department of Biomedical Engineering The University of Texas at Austin Austin Texas USA

**Keywords:** high‐frequency ultrasound, nanodroplets, perfluorocarbon contrast agents, ultrasound activation

## Abstract

**Background:**

While microbubble contrast agents (MCAs) are commonly used in ultrasound (US), they are inherently limited to vascular targets due to their size. Alternatively, phase‐changing nanodroplet contrast agents (PNCAs) can be delivered as nanoscale agents (i.e., small enough to extravasate), but when exposed to a US field of sufficient mechanical index (MI), they convert to MCAs, which can be visualized with high contrast using nonlinear US.

**Purpose:**

To investigate the effect of perfluorocarbon (PFC) core composition and presence of cholesterol in particle coatings on stability and image contrast generated from acoustic activation of PNCAs using high‐frequency US suitable for clinical imaging.

**Methods:**

PNCAs with varied core compositions (i.e., mixtures of perfluoropentane [C5] and/or perfluorohexane [C6]) and two coating formulations (i.e., with and without cholesterol) were characterized and investigated for thermal/temporal stability and postactivation, nonlinear US contrast in phantom and in vivo environments. Through hydrophone measurements and nonlinear numerical modeling, MI was estimated for pulse sequences used for PNCA activation.

**Results:**

All PNCA compositions were characterized to have similar diameters (249–267 nm) and polydispersity (0.151–0.185) following fabrication. While PNCAs with majority C5 core composition showed higher levels of spontaneous signal (i.e., not due to US activation) in phantoms than C6‐majority PNCAs, all compositions were stable during imaging experiments. When activating PNCAs with a 12.3‐MHz US pulse (MI = 1.1), C6‐core particles with cholesterol‐free coatings (i.e., CF‐C6‐100 particles) generated a median contrast of 3.1, which was significantly higher (*p *< 0.001) than other formulations. Further, CF‐C6‐100 particles were activated in a murine model, generating US contrast ≥3.4.

**Conclusion:**

C6‐core PNCAs can provide high‐contrast US imaging with minimal nonspecific activation in phantom and in vivo environments.

## 1 INTRODUCTION

While several groups have investigated the use of microbubble contrast agents (MCAs) as molecular ultrasound (US) imaging targets, their size does not allow for vascular extravasation (e.g., via the enhanced permeability and retention effect), limiting them to intravascular delivery.^1^ Although nanoscale agents are necessary for extravascular delivery, enhanced US contrast is achieved by MCAs due to their gaseous core and micron‐order size, resulting in a resonance frequency within typical diagnostic US bandwidths.^2^ This discrepancy in optimal agent properties for vascular extravasation and US imaging has led to the development of perfluorocarbon (PFC)‐cored phase‐changing nanodroplet contrast agents (PNCAs).^3^, ^4^, ^5^ PNCAs share a similar chemical makeup to MCAs, but they are designed to be stored and administered as liquid‐core nanodroplets rather than as gaseous microbubbles to facilitate extravasation and cellular uptake.^4^ However, once PNCAs have been extravascularly delivered, negative pressure from focused US pulses can be used to locally “activate” them, converting the nanodroplets into gas‐cored microbubbles for high‐contrast US imaging.^3^, ^4^, ^5^ As the necessary activation threshold (aT) has been shown to depend on nanodroplet size and core composition, modifications to PNCA formulations have been investigated to simultaneously optimize particle stability (i.e., to prevent spontaneous activation and improve shelf life) and US contrast.^3^, ^6^

When thermally stable particles with minimal nonspecific activation are desired, PNCAs are generally formulated with relatively high‐boiling‐point (HBP) PFC cores (i.e., higher aT), such as perfluoropentane, C_5_F_12_ (C5; BP: 29°C), and perfluorohexane, C_6_F_14_ (C6; BP: 56°C).^7^ In addition to the PFC core, changes in a PNCA's lipid coating have also been investigated in an attempt to increase their stability.^8^, ^9^ However, due to the higher aT inherent to these stable particles, higher‐pressure transmits are necessary for their activation, thereby increasing the risk of producing harmful bioeffects (e.g., hemolysis and capillary endothelial injury^10^). Therefore, mechanical index (MI), defined as $\frac{P_{N}}{\sqrt{f_{c}}}$, where *P_N_* is peak negative pressure [MPa] and *f_c_* is the ultrasonic center frequency [MHz], is evaluated for such transmits to help ensure patient safety, with the US Food and Drug Administration (FDA) permitting MI ≤1.9 for diagnostic imaging.^6^ Use of high‐frequency US presents unique benefits for activation and imaging of PNCAs because of MI's inverse proportionality with *f_c_* (i.e., allowing higher transmit pressures), lower associated aT due to harmonic focusing within the droplets,^11^ and improved image resolution.

Although therapeutic US activation sequences (e.g., high‐intensity focused US^12^) and laser activation of optically loaded PNCAs with (e.g., sono‐photoacoustic imaging^13^) and without acoustic activation^14^ have proven successful despite potential safety and penetration‐depth limitations, there has been minimal success to date in the activation of C6‐core PNCAs with diagnostic US due to their relatively high aT. We address this problem by investigating high‐frequency (i.e., 12.3 MHz) activation and imaging of PNCAs with varying HBP PFC cores and two distinct lipid shells to achieve a stable formulation that can be reliably activated and imaged with high contrast using diagnostic US.

## 2 MATERIALS AND METHODS

Five PNCA formulations with different PFC cores (100% C5 [C6‐0]; 75% C5 and 25% C6 [C6‐25]; 50% C5 and C6 [C6‐50]; 25% C5 and 75% C6 [C6‐75]; and 100% C6 [C6‐100]) were prepared according to previous work,^15^, ^16^, ^17^, ^18^ with PFC mixtures being mixed on a per‐particle level. Briefly, a lipid cake composed of 18.0 mg 1,2‐distearoyl‐sn‐glycero‐3‐phosphocholine (DSPC, 94.3% by molarity), 1.7 mg 1,2‐distearoyl‐sn‐glycero‐3‐phosphoethanolamine‐N‐methoxy‐polyethyleneglycol‐2000 (DSPE‐PEG‐2000, 2.5%), and 0.3 mg cholesterol (3.2%; all from Avanti Polar Lipids, Inc., Alabaster, AL) was formed and then rehydrated in 2 ml deionized (DI) water for each of these preparations. One additional preparation (CF‐C6‐100) was made with 100% C6 but without cholesterol (i.e., cholestrol‐free [CF]) using the following composition: 18.6 mg DSPC (97.5%) and 1.7 mg DSPE‐PEG‐2000 (2.5%). Next, 200 μl of each PFC core material was mixed with 100 μl 1% (v/v) aqueous solution of 1H,1H,2H‐Perfluoro‐1‐hexene,3,3,4,4,5,5,6,6,6‐nonafluoro‐1‐hexene (ZONYL® PFBE; Sigma‐Aldrich Corp., St. Louis, MO) and 150 μl ice‐cold DI water under vortexing, followed by bath sonication (CPX‐962‐218R; Thermo Fisher Scientific, Waltham, MA) in ice‐cold water for 30 s. ZONYL® PFBE was added to facilitate emulsification of PFCs. Note that in contrast to previously reported ZONYL® FSO^19^, ^20^ and ZONYL® FSP^21^, ^22^ fluorosurfactants, ZONYL® PFBE has a much shorter hydrocarbon chain (i.e., one carbon atom), which is not sufficient for a stable emulsification of a PFC core material. The rehydrated lipid cake was then added to vials with PFC core materials, vortexed for 30 s, and then sonicated in ice‐cold water for 1 min. Finally, each suspension was sonicated with a 2‐mm‐tip probe‐sonicator (VCX 500; Sonics & Materials, Inc., Newtown, VT) for two 1‐min cycles at 25% maximum amplitude, separated by 20 s of vortexing. A DelsaNano C dynamic light scattering system (Beckman Coulter, Inc., Brea, CA) was used to measure the mean size and polydispersity index (PDI) of nanodroplets in triplicate approximately 1 and 8 weeks after fabrication (i.e., before and after all US characterization). The relative amount of PFC in stock nanodroplet preparations was measured using ^19^F nuclear magnetic resonance (NMR), as previously described.^15^ Briefly, NMR scans were carried out using a 500‐MHz NMR spectrometer (Bruker Corporation, Billerica, MA), and ^19^F concentrations were determined by integrating C5 and/or C6 NMR peaks with normalization by the integrated peak of the known trifluoroacetic acid standard. Aqueous PNCA stock preparations were stored in sealed glass vials at 4°C and mixed by gentle vortexing for 5–10 s before each use.

**FIGURE 1:**
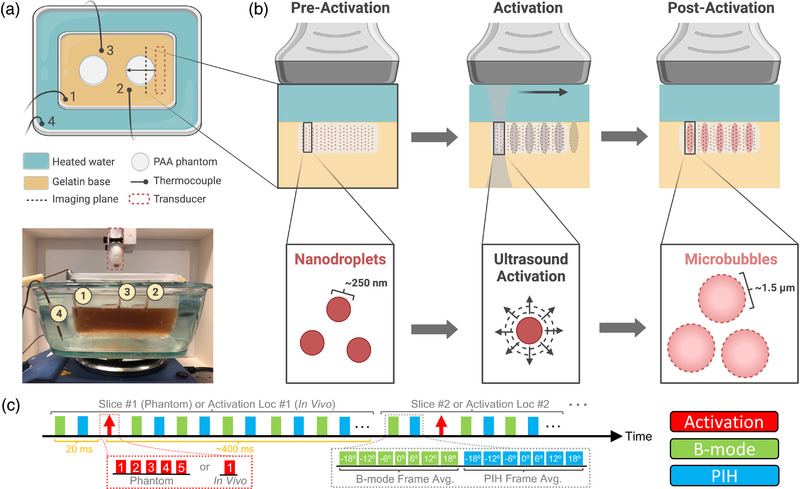
Phase‐changing nanodroplet contrast agents (PNCAs) activation in a phantom at 37°C. (a) Overhead diagram (top) and side‐view photograph (bottom) of phantom imaging setup atop hot plate with translation‐stage‐mounted ultrasound (US) transducer above. (b) Imaging and activation process in one slice of PNCA‐containing polyacrylamide (PAA) phantom (light gray rectangle). Preactivation (left), phantom is uniform with nonactivated PNCAs (dark red circles). During activation (center), PNCAs coinciding with “activation” pulse (dark gray “hourglass” with ovular focal spots) transition into gas‐filled microbubble contrast agents (MCAs) (larger, pink circles), as seen postactivation (right). PNCAs not coinciding with activation pulses (e.g., phantom regions outside pink ovals) remain intact. (c) Imaging and activation timing diagram (above) for sequences used in phantom and in vivo studies. Note values in red and green/cyan boxes within insets (below) indicate activation location number or plane‐wave steering angle (i.e., 0° = transducer normal), respectively.

A Vantage 128 US system (Verasonics Inc., Kirkland, WA) with an L22‐8v CMUT linear array (15‐MHz *f_c_*, 15‐mm elevation focus; Kolo Medical Co., Ltd., Suzhou, China) mounted to a BiSlide translation stage (Velmex Inc., Bloomfield, NY) was used for imaging and activation (sequence shown in Figure 1c), which consisted of co‐registered plane‐wave B‐mode and pulse inversion harmonic (PIH) imaging ensembles (9‐MHz *f_c_*, 7‐angle average, 50‐Hz frame rate) acquired before and after focused activation pulses (12.3‐MHz *f_c_*, 0.9 F/#, 6 cycles). To estimate the MI for activation pulses, MATLAB‐based linear acoustic simulation software, Field II,^23^ was first used to model the normalized pressure resulting from an activation transmit (active aperture: 13.8 × 2.5 mm) in a region (20 × 6 mm; 0.1‐mm and 250‐MHz sampling) centered about the array surface 2 mm away axially. A 0.2‐mm NH0200 needle hydrophone positioned 2 mm from the L22‐8v array face in a degassed UMS Research water tank (all from Precision Acoustics Ltd., Dorchester, UK) was then used to measure the peak‐positive pressure resulting from activation transmits at points within a centered subsample (5 × 1 mm; 1/0.5‐mm and 250‐MHz sampling) of the modeled region. To generate a pressure‐scaling factor, the spatial average of these measurements was then divided by a region‐matched spatial average of the peak‐positive Field II result. This pressure‐scaled distribution (i.e., scaling factor x original field II result) at 2 mm depth was finally imported into MATLAB‐based nonlinear acoustic modeling software, Fullwave^24^ and propagated through 11 mm of water (*α *= 0.0022 dB/cm/MHz; *β *= 3.5)^25^ and 2 mm of polyacrylamide (PAA) (*α *= 0.15 dB/cm/MHz; *β *= 3.5)^26^ to the 15‐mm focus, resulting in an estimate of *P_N_* (MPa) for calculation of MI in the phantom setup.

For phantom studies, aliquots from the six PNCA stock preparations were first diluted in phosphate‐buffered saline to have matched ^19^F concentrations of 40 mM. These PNCA dilutions were then combined with PAA and cast in a six‐well cell culture plate to make phantoms composed of 1000 μl of 40% (w/v) acrylamide/bis 19:1 solution, 60 μl of ammonium persulfate (440 mM aqueous solution), 8 μl of tetramethylethylenediamine (all from Sigma‐Aldrich Corp.), 30 μl of nanodroplet dilution, and 3,000 μl of DI water.^27^ Using cyanoacrylate glue (HR4‐316; Thermo Fisher Scientific), phantoms were then secured to a gelatin (C9382; Sigma‐Aldrich Corp.) base cast in a plastic container, which was filled with DI water and put on a plastic stand (i.e., allowing for water circulation) inside an empty water bath placed atop a hot plate (below image in Figure 1a). The water bath was then filled with 60°C water, and the hot plate was adjusted to achieve a phantom temperature of ∼37°C. A TC‐08 data logger (Omega Engineering Inc., Norwalk, CT) recorded temperature every 10 s using four K‐type thermocouple leads (Milwaukee Tool Corp., Brookfield, WI), with one placed in the water bath, and three placed in the gelatin base adjacent to PNCA‐containing phantoms (above schematic in Figure 1a).

**FIGURE 2:**
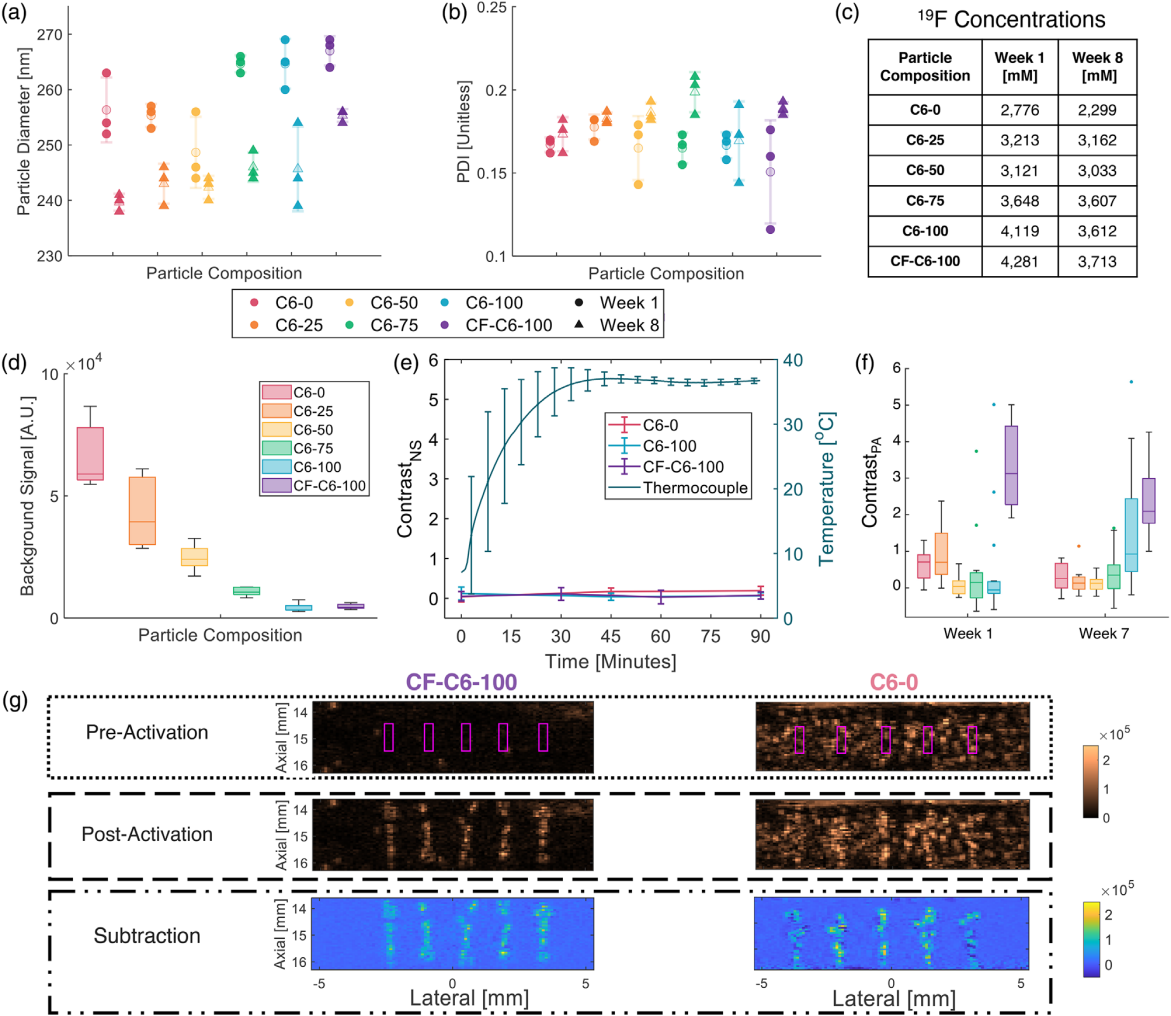
Characterization of phase‐changing nanodroplet contrast agents (PNCAs) size, stability, and activatability. (a) Dynamic light scattering (DLS)‐based size and (b) polydispersity index (PDI) distributions (data points shown over mean ± standard deviation [SD] transparency) for all compositions week 1 (circle) and week 8 (triangle) after fabrication. Note legend applies to both plots. (c) ^19^F concentrations for stock nanodroplet preparations at weeks 1 and 8. (d) Box plots (i.e., median, 25th/75th quartiles, and 1.5x interquartile range) of background signal 1 week after fabrication (*n* = 5) for all formulations with color‐coded legend, which also applies to (e and f). (e) Plots (mean ± SD) of contrast_NA_ (*n* = 5; left axis) and average thermocouple temperature (*n* = 3; right axis) versus time in C6‐0, C6‐100, and CF‐C6‐100 phantoms. (f) Box plots (outliers shown) of contrast_PA_ (*n* = 15) for all PNCA compositions at week 1 (left) and week 7 (right). (g) Representative preactivation (top), postactivation (middle), and corresponding subtraction images (bottom) for CF‐C6‐100 (left) and C6‐0 (right) PNCAs at week 1, with magenta boxes denoting activation locations and contrast_PA_ ROIs. Same dynamic range (shown far right) used for matched imaging modes.

Six PAA phantoms, each containing a different PNCA composition, were constructed to test PNCA background signal (i.e., spontaneous activation) and activatability. Background signal was assessed with PIH imaging of phantoms immediately prior to water‐bath filling, while activatability was assessed once phantoms reached ∼37°C with PIH imaging immediately before and ∼400 ms after the last of five activation pulses (15‐mm focus) spaced 1–2 mm apart (Figure 2g). Imaging and activation sequences were repeated across three elevational slices (1‐mm spacing), providing 15 unique activation locations. To track changes in PNCA activation due to prolonged storage, new PAA phantoms containing PNCAs from each of the six preparations were constructed 7 weeks after PNCA fabrication and imaged using the same protocol.

Background signal, $\bar{S_{t<0}}$, was calculated at *t *< 0 (i.e., prior to water‐bath filling) as the mean signal in one slice of the PIH imaging data within each of five regions of interest (ROIs; 0.32 × 0.9 mm) centered laterally with 2‐mm spacing at 15‐mm depth. Postactivation image contrast, contrast_PA_, was calculated as $\frac{\bar{S_{A}}-\bar{S_{B}}}{\bar{S_{B}}}$, where $\bar{S_{A}}$ is the mean signal within each ROI (0.32 × 0.9 mm; see Figure 2g) centered about an activation, and $\bar{S_{B}}$ is the mean signal in a paired (i.e., same size/depth but laterally offset 0.53 mm) background ROI. Subtraction images were generated by subtracting postactivation PIH images from their matched preactivation counterpart. Significance (i.e., indicating *p *< 0.05 whenever stated) of inter‐week (i.e., fixed formulation between two time‐points) and intra‐week (i.e., fixed time across formulations) differences was assessed using a two‐tailed, two‐sample, unpaired *t*‐test, and one‐way ANOVA with post hoc Tukey testing, respectively.

To test PNCA temporal and thermal stability, phantoms with the least (C6‐0) and most (C6‐100 & CF‐C6‐100) stable formulations (i.e., having the highest or lowest background signals [see Figure 2d], respectively) were fabricated, and PIH imaging (i.e., no activation pulses) of these phantoms was conducted immediately after water‐bath filling (i.e., *t *= 0) and repeated for 90 min. Nonspecific contrast (contrast_NS_) was then calculated at each time‐point as $\frac{\bar{S_{t}}-\bar{S_{t<0}}}{\bar{S_{t<0}}}$, where $\bar{S_{t}}$ is the mean signal within the same ROIs as previously defined for $\bar{S_{t<0}}$ but for *t* ≥0. Nonspecific activation resulting from PNCA injections was also assessed ex vivo in degassed porcine skeletal muscle at a depth of ∼15 mm using 27‐ and 25‐gauge needles prior to in vivo imaging (i.e., bolus injection followed by PIH imaging). Contrast_NS_ was calculated, as previously described, with time‐points immediately before and after injection but using only a single ROI centered about the injection region, which was identified with B‐mode US. Significance for contrast_NS_ results was assessed with a one‐way ANOVA with post hoc Tukey testing.

**FIGURE 3:**
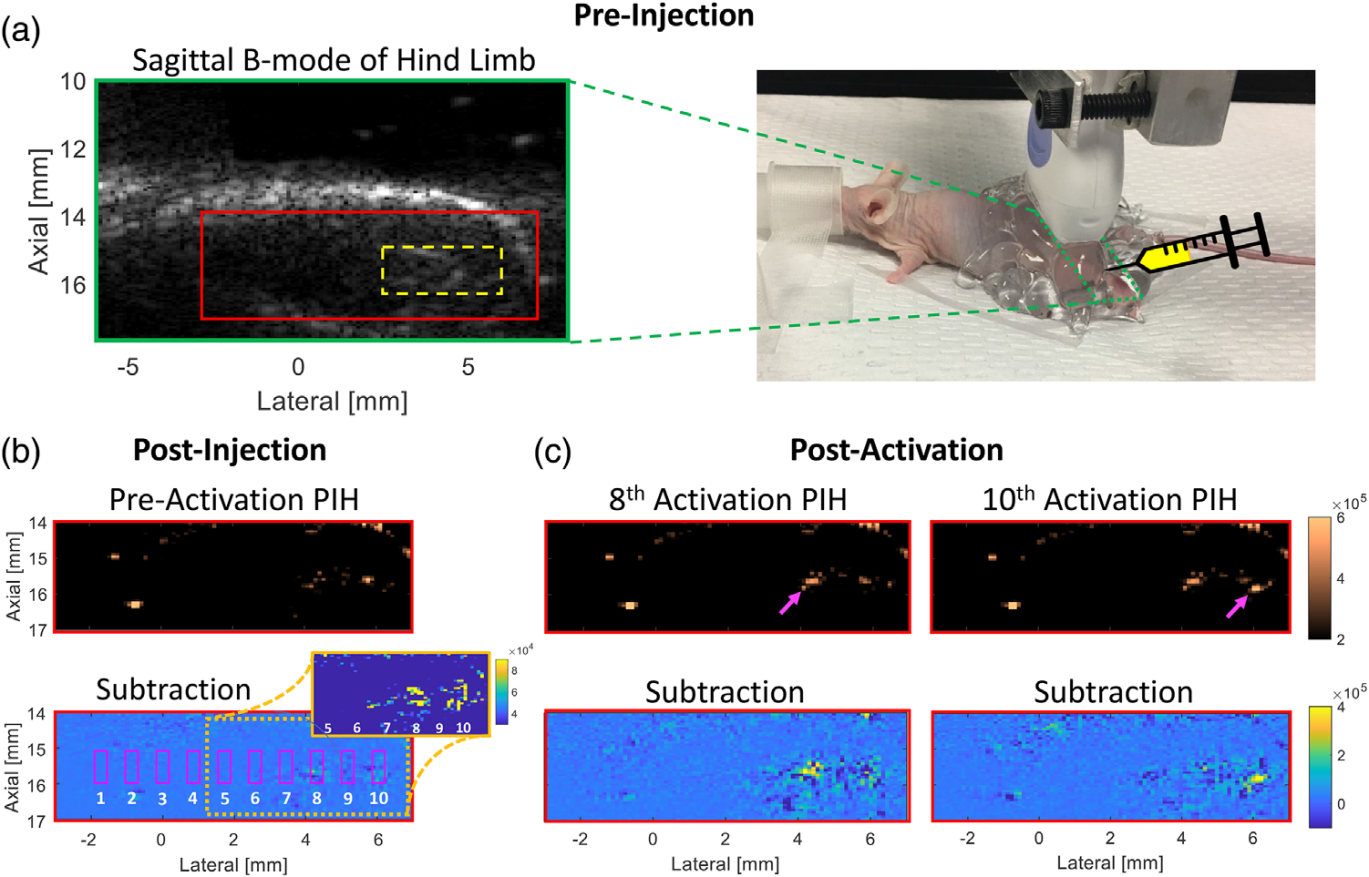
In vivo phase‐changing nanodroplet contrast agents (PNCAs) activation and imaging. (a) Preinjection B‐mode image (left) with injection region noted (dashed yellow box) and picture of experimental setup (right) with transducer (moved for visibility) footprint noted (dashed green box) for sagittal imaging of hind limb. (b) Postinjection, preactivation pulse inversion harmonic (PIH) (top), and subtraction (bottom) images, with the latter indicating numbered activation locations/ROIs (magenta boxes). Because injection bolus is not apparent in subtraction image, an image inset with reduced dynamic range is included to clearly show preactivation injection region (yellow at 4–6 mm laterally). (c) PIH (top) and subtraction (bottom) images showing focal contrast enhancement that is temporally/spatially coincident with 8th (left) and 10th (right) activations (magenta arrows) and within preactivation injection region. Same dynamic range used for matched imaging modes.

Finally, an in vivo proof‐of‐concept study was performed in an 8‐week‐old athymic nude male mouse in compliance with MD Anderson Cancer Center's Institutional Animal Care and Use Committee. During imaging, the mouse was anesthetized (maintained 2.5% isoflurane; 1 L/min O_2_) and placed on a heating pad to maintain adequate core temperature (right Figure 3a); respiration rate was visually monitored. The mouse's hind limb was first imaged without activation (B‐mode and PIH) to obtain a baseline and identify an injection site. Informed by the in vitro injection experiment, a 20‐μl intramuscular (IM) injection of 3300 mM CF‐C6‐100 PNCA was performed in the hind limb using a 25‐gauge needle with US guidance to ensure an injection depth of 15–16 mm. A postinjection imaging baseline was then acquired, followed by a modified phantom imaging‐activation sequence, which acquired PIH imaging data in one slice for ∼400 ms after each of 10 activations (15.5‐mm focus) spaced 0.8 mm apart. The injection location was confirmed by mild enhancement observed in the postinjection, preactivation PIH subtraction image (inset in Figure 3b). The mouse was euthanized with CO_2_ inhalation and cervical dislocation following imaging. Contrast_PA_ and contrast_NS_ were calculated for all activation sites.

## 3 RESULTS

One week after fabrication, PNCA compositions had mean diameters ranging from 249 to 267 nm (Figure 2a), with PDI from 0.151 to 0.185, indicating good homogeneity (Figure 2b). At 8 weeks, diameters significantly (i.e., *p *< 0.05) decreased in all samples (maximum decrease of 15 nm) except for C6‐50 (*p *= 0.18), while PDI did not significantly change for any sample other than C6‐75 (*p *= 0.022). There was a decrease in ^19^F sample concentrations (Figure 2c) ranging 1%–17% from 1 to 8 weeks, indicating good stability of the formulations during storage.

PNCAs exhibited a monotonic decrease in background (i.e., spontaneous and nonactivated) US signal (Figure 2d) as C5 percentage in the core mix decreased. Contrast_NS_ (Figure 2e) did not significantly change (*p* ≥ 0.28) over 90 min during or after temperature acclimation for the most stable formulations (i.e., C6‐100 and CF‐C6‐100), while a significant increase (*p *= 0.037) through time was observed for C6‐0. The average temperature within the gelatin base (i.e., surrogate for phantom temperature) reached the 37°C setpoint at ∼45 min and remained at 37+/−1°C for more than 45 min.

Based on nonlinear modeling initialized with transducer‐surface hydrophone measurements, *P_N_* was estimated to be 3.7 MPa for the activation transmit (15‐mm focus), yielding an MI of 1.1. Using these activation pulses on PNCAs 1 week after particle fabrication, C6‐0 (right Figure 2g) and C6‐25 PNCAs both provide median contrast_PA_ of 0.8 (Figure 2f), while other cholesterol‐containing formulations failed to provide reliable contrast. In comparison, the cholesterol‐free CF‐C6‐100 (left Figure 2g) formulation yielded a significantly higher contrast_PA_ of 3.1 (Figure 2f; *p *< 0.001). Following 7 weeks, majority‐C5 PNCA formulations presented with significant decreases in contrast_PA_ (*p *= 0.026 and 0.001 for C6‐0 and C6‐25, respectively) as did CF‐C6‐100 (to 2.2; *p *= 0.019), while C6‐50, C6‐75, and C‐100 PNCAs experienced no significant change (*p* ≥0.054).

Injection of PNCAs with a 27‐gauge needle in ex vivo tissue resulted in a contrast_NS_ (i.e., no activation) of 3.6, while injection with a 25‐gauge needle yielded a contrast_NS_ of only 0.7, identifying the latter as best suited for mouse imaging. In vivo activation of PNCAs was clearly visible in PIH imaging (top Figure 3c). Contrast enhancement was spatially and temporally coincident with the injected region (i.e., yellow in Figure 3b subtraction inset) and activation‐pulse order, respectively, presenting first at activation #8 (left Figure 3c; contrast_PA_ = 3.4), and then later at activation #10 (right Figure 3c; contrast_PA_ = 3.6). The maximum contrast_PA_ for all other activations was 0.6, while a maximum contrast_NS_ of 0.9 was observed postinjection but preactivation.

## 4 DISCUSSION

This work demonstrates that HBP‐core PNCAs can be reliably activated with MI below FDA limits and precise spatiotemporal control using high‐frequency diagnostic US sequences in both phantom and in vivo environments. PAA‐based phantoms allow for precise and stable imaging and activation of PNCAs at body temperature. PNCAs with a predominantly C5 core appear to spontaneously activate during phantom fabrication due to the exothermic reaction of PAA cross‐linking, contributing to increased background signal (Figure 2d).

While the three PNCA compositions with core mixtures of both C5 and C6 provided thermal stability that was approximately the average of their constitutive C5/C6 components, yielding background signal (Figure 2d) that monotonically decreased with increasing C6 percentage in the core mix, they failed to yield median contrast_PA_ >0.8, as shown in Figure 2f. Although C6‐0 and C6‐25 were readily activatable, particularly at week 1, they yielded reduced contrast_PA_ due to their increased nonspecific activation (Figure 2d) and the resulting increase in preactivation signal. In comparison, C6‐50 and C6‐75 at both time‐points (i.e., weeks 1 and 7) and C6‐100 at week 1 generated relatively low levels of nonspecific activation, but they appeared to maintain prohibitively high aT, which resulted in relatively low overall contrast_PA_. Interestingly, C6‐100 experienced an increase (*p *= 0.054) in contrast_PA_ at week 7. Since we did not observe any significant change in the size of C6‐100 nanodroplets over time, we hypothesize that this decrease in aT is likely associated with changes in the PFC core/lipid interface and/or the lipid coating. It is important to note that US signal enhancement, which directly affects contrast, depends on resulting microbubble size (i.e., resonance frequency), which can be impacted by intra‐droplet/bubble dynamics such as coalescence.^28^ The importance of lipid coating composition is supported by the low aT of cholesterol‐free PNCAs (CF‐C6‐100) as they provided maximum contrast_PA_ fresh after preparation (purple in Figure 2f) while maintaining sufficient environmental stability to also yield minimal nonspecific activation (purple in Figure 2d).

Injection of PNCAs into tissue can result in nonspecific activation, an effect that appears to increase with decreasing needle size and is likely due to changes in hydrostatic pressure encountered during the injection.^29^ The impact of injection rate and whether this effect occurs during intravascular injection for systemic delivery must be investigated in future work. Although CF‐C6‐100 PNCAs presented with modest nonspecific activation following IM injection (e.g., slight hyperechoic, preactivation signal at activation locations in top Figure 3b), this work demonstrates the ability to specifically activate C6‐core PNCAs in an in vivo environment. Future work will explore the optimization of PNCA coatings to ensure that samples are readily activatable with minimal spontaneous activation for continued in vivo translation. Additionally, PNCAs with functionalized lipids will be conjugated to antibodies to investigate targeting efficacy.

## 5 CONCLUSIONS

This work demonstrates the effect of changing core material and presence of cholesterol on stability and activatability of PNCAs. C6‐majority PNCAs are more resistant to spontaneous activation than C5‐majority PNCAs, leading to low contrast_PA_ values for all C5‐majority particles. While C6‐core PNCAs also showed limited contrast_PA_ 1 week after fabrication, time‐dependent changes (i.e., storage for 7 weeks) and modification to a cholesterol‐free lipid coating (i.e., CF‐C6‐100) cause a decrease in aT and subsequent increase in contrast_PA_. Additionally, although CF‐C6‐100 particles showed low‐level activation upon in vivo injection, they presented strong contrast enhancement that was spatiotemporally coincident with high‐frequency activation pulses, indicating their potential as stable PNCAs that can be activated with diagnostic US sequences.

## CONFLICT OF INTEREST

The authors declare that there is no conflict of interest related to this work.
